# A Novel Hybrid Chaotic Map and Cryptographic Hash Whitening for Optimized S-Box Design: Construction, Cryptanalysis, and Lightweight IoT Sensor Evaluation

**DOI:** 10.3390/s26134316

**Published:** 2026-07-07

**Authors:** Nahar F. Alshammari, Faraj H. Alyami, Abdullah G. Alharbi, Saleh Al Dawsari, Yousaf Hameed Khattak, Faisal Baig

**Affiliations:** 1 Department of Electrical Engineering, Faculty of Engineering, Jouf University, Sakaka 72388, Saudi Arabia; nfalshamari@ju.edu.sa; 2 Electrical Engineering Department, College of Engineering, Najran University, Najran 11001, Saudi Arabia; fhualyami@nu.edu.sa; 3 Science and Engineering Research Center, Najran University, Najran 11001, Saudi Arabia; 4 Department of Electrical Engineering, College of Engineering, Princess Nourah Bint Abdulrahman University, P.O. Box 84428, Riyadh 11671, Saudi Arabia; abgalharbi@pnu.edu.sa; 5 School of Engineering, Cardiff University, Cardiff CF24 3AA, UK; 6 School of Design Engineering, Universitat Politecnicia de Valencia, 46022 Valencia, Spain; engr.fsl.baig@gmail.com; 7 Software Engineering Department, Federal Urdu University of Arts, Science & Technology, Islamabad 45570, Pakistan

**Keywords:** hybrid chaotic systems, S-box design, nonlinear dynamics, cryptographic security, image encryption

## Abstract

**Highlights:**

**What are the main findings?**

- A hybrid S-box design was developed by combining delayed nonlinear chaotic dynamics, SHA-256-based hash whitening, Fisher–Yates permutation, and affine transformation over GF (2), producing high-quality 8-bit → 8-bit S-boxes displayed in 16 × 16 hexadecimal format.
- The optimized S-box showed strong cryptographic performance, including a nonlinearity of 104, differential uniformity of 10, avalanche effect near the ideal value (50.6%), algebraic degree of 7, and successful image-encryption validation.

**What are the implications of the main findings?**

- The proposed hybrid chaotic–hash framework improves resistance against linear and differential cryptanalysis, making it a promising candidate for secure symmetric cryptographic applications.
- The successful grayscale image-encryption test indicates that the designed S-box is not only theoretically strong but also practically useful for multimedia and data-security systems.

**Abstract:**

In this work, a novel method was introduced for the construction of the S-box based on delayed nonlinear chaotic systems coupled with a hybrid hash whitening mechanism. The evidence for strong nonlinear dynamical behavior is the strongly positive Lyapunov exponent with uniform statistical distribution of the generated binary sequence. Optimized 8-bit → 8-bit S-boxes, displayed in 16 × 16 hexadecimal format, were achieved by applying Fisher–Yates permutation to the chaotic sequences and further enhancing them with affine transformations over GF(2). Nonlinearity, differential uniformity, avalanche effect, strict avalanche criterion, linear approximation table, difference distribution table, and algebraic degree are cryptographic metrics that reveal very strong resistance against linear and differential attacks. Image encryption with the generated S-box further validates the confusion properties. The results confirm that the hybrid approach achieves high-quality S-boxes suitable for symmetric cryptography.

## 1. Introduction

In this emerging digital world where technology is swiftly upgrading, information security has gained new importance at present [1]. Due to the increased usage of communication and data transfer via the Internet, security must be increased regarding sensitive information [2]. Cryptography plays a crucial role in ensuring confidentiality, integrity, and authentication of information. It grants secure communication over unsecured networks by encoding and decoding the information so that it cannot be accessed or intercepted by unauthorized sources [3,4].

In the war of data security, symmetric cryptosystems, first introduced by Shannon in 1949, remain a cornerstone in the implementation of these security protocols. They rely considerably on two foundational concepts: confusion and diffusion [5]. Confusion involves complex and unpredictable rearrangement of data, such as the pixels in images, in order to mask the inherent relationship between plaintext and ciphertext. Diffusion, however, ensures that the variation in a single element of plaintext is spread widely within the entire cipher [2,5]. Block ciphers, encryption methods that divide their plaintext into fixed-size blocks and then process them using specific algorithms, using both confusion and diffusion, produce cipher texts that are practically impossible to decipher without the right key [6,7]. The most critical element in designing block ciphers is the substitution box, S-box, which acts as the major nonlinear component and hence carries the responsibility for the cryptographic strength of the cipher [8]. S-boxes introduce nonlinearity and confusion into the encryption process through the conversion of input bits into completely different output bits. This nonlinear transformation is required for providing resistance against some powerful cryptanalytic attacks like differential and linear cryptanalysis, due to the fact that it masks patterns and relationships in the encrypted data [9].

Despite their importance, many S-box construction approaches may exhibit weaknesses such as fixed points (S(x) = x), reverse fixed points (S(x) = x′), and short-period cycles, which may allow ciphers to be vulnerable to statistical attacks. Notably, algebraically constructed S-boxes such as the AES S-box are explicitly designed to avoid these issues: the AES S-box has zero fixed points, zero reverse fixed points, differential uniformity DU = 4, and nonlinearity NL = 112. In contrast, purely chaos-based and heuristically optimized S-boxes often require additional refinement to suppress such weaknesses [10]. Constructing optimal S-boxes from a cryptographic viewpoint is, however, considered to be a complex combinatorial problem because the search space is extremely large, no guidelines are available, and there exist conflicting criteria for their design [11]. For instance, an (8, 8)-function S-box allows as many as (2^8^)! or 256! possible bijective mappings, which gives an idea about the computational issue involved [12]. Generally speaking, approaches to S-box construction fall into several broad categories.

*Algebraic Constructions*: These include mathematical structures like Galois field theory, affine transformations, and inverse mappings. Some well-known examples are the AES S-box, which is an (8, 8)-function based on Galois field inversion and affine mapping. Other techniques include Mübius transformations [13,14,15], CFT or cubic fractional transformation [16], LFT or linear fractional transformation with Gaussian distribution [17,18], cellular automata [19], complete Latin squares [7], linear trigonometric transformations [20,21,22], and the use of cyclic and symmetric groups [14,23,24]. Algebraic methods generally provide superior nonlinearity and good cryptographic properties [25]. However, they also have some drawbacks, such as limited key space, the presence of fixed and reverse fixed points, short-period cycles, and vulnerability due to algebraic attacks because of hidden algebraic structures [26,27,28,29]. In addition, these techniques usually generate only one S-box per function. Very seldom does any new fractional transformation come into consideration [28,30].

*Chaos-Based Constructions*: Chaos theory, with its nonlinear dynamics, unpredictability, high randomness, and sensitivity to initial conditions, is widely employed in cryptography [7,31]. Numerous chaotic systems, including 1D [32], 2D [17,33], hyperchaotic [34] and fractional-order maps [35], have been introduced into S-box design [36]. Examples include discrete chaotic maps [37], the Lorenz map [38], improved logistic maps [39], chaotic maps combined with quantum walks [40], fractional-order Chen systems [41], the Gingerbread man map [3,42] and the tent–sine (TS) chaotic system [18,43,44]. Chaos-based dynamic S-boxes are key-dependent, yield different outputs, improve the cipher strength, and also have a large key space [4,8,45]. However, they usually exhibit either low nonlinearity or high computational complexity due to the dimensionality of chaotic mappings [19,26,27]. Moreover, low-dimensional chaos has unwanted chaotic behavior, which is often coupled with restricted ranges of its chaotic behavior, thus introducing weak encryption properties [44,46].

*Heuristic and Optimization Methods*: These techniques employ algorithms like GA [17,26,47], artificial bee colony (ABC) [26,47], cuckoo search [26], firefly algorithm [26], whale optimization, and sine–cosine optimization [26] to search through the space for S-boxes with the best properties. Other heuristic approaches include BFO [48], AJO [49], GWO [50], simulated annealing, and hill climbing [26]. These algorithms make use of fitness functions and can produce a varied set of S-boxes that do not have inherent algebraic structures [14]. Though in general this approach yields better S-boxes than random generation and metrics such as nonlinearity can be optimized [24], the cryptographic strength of these S-boxes may not be comparable to algebraic methods and solution quality guarantees are also not available [14].

*Hybrid Construction Methods (Emerging Trend)*: To overcome the limitations of each approach in isolation, various hybrid S-box construction methods have gained increasing popularity [16,17,23]. These include methods that combine chaotic systems with algebraic mappings or algebraic constructions enhanced through heuristic algorithms, thus taking advantage of their complementarity [27,51]. For example, the chaotic opposition-based learning initialized hybrid algebraic–heuristic (COBLAH) algorithm combines Galois field inversion, affine mapping, and GA to generate S-boxes possessing superior cryptographic characteristics of high nonlinearity, low differential uniformity, and large key space while reducing weaknesses such as fixed points and short cycles [7].

The literature review showed that a hybrid approach gives a more secure and nonlinear S-box. Inspired by this trend, we introduce in the present work a hybrid chaotic–hash whitening framework where delayed nonlinear chaotic dynamics are coupled with cryptographic hash whitening. The so-produced sequences undergo refinement via Fisher–Yates permutation and affine transformations over GF (2) for obtaining optimized S-boxes displaying superior nonlinearity and avalanche features along with robustness against various cryptanalytic attacks. Finally, practical robustness of the obtained S-boxes is further established over an image encryption task which justifies their validity for use in contemporary symmetric cryptography. The distinct novelty of the presented approach lies in the co-design of three components:

*Novelty 1: Delay-Embedded Nonlinear Chaotic Map*: A delayed nonlinear map (Equations (1) and (2)) with tunable coupling parameter ε introduces multi-step memory into the trajectory, preventing the short-period orbits common in standard 1D logistic or tent maps and increasing the Lyapunov exponent to λ = 17.496, confirming strong chaotic dynamics.

*Novelty 2: Inline SHA-256 Hash Whitening*: SHA-256 whitening is embedded into the sequence-generation pipeline (not applied as post-processing) by XOR-combining each 256-bit chaotic block with its SHA-256 hash. This corrects finite-precision statistical artifacts before S-box candidate construction, enabling the whitened sequence to pass all NIST SP 800-22 tests including the Random Excursion test.

*Novelty 3: Three-Criterion Constrained S-Box Selection*: An 80-trial optimization loop enforces hard cryptographic constraints (zero fixed points, zero 2-cycles, zero reverse fixed points) and selects the best candidate by NL → DU → avalanche priority, yielding NL = 104, DU = 8, AI = 4 (all coordinates), algebraic degree = 7, and confirmed deployment at 126.58 KB/s on Arduino Uno.

The approach combines chaotic and hash-optimization schemes, thereby distinguishing the proposed method from traditional purely chaos-based, purely algebraic, and entirely heuristic-only S-box construction schemes [8,29,47].

## 2. Proposed Method

The proposed approach consists of three key phases: delayed nonlinear chaotic sequence generation, whitening and binary sequence formation using the SHA-256 hash function, and S-box optimization using the Fisher–Yates permutation and affine transformation over GF(2). The main objective of this structure is to combine chaotic sensitivity with cryptographic processing and controlled S-box selection.

*Delayed Nonlinear Chaotic Map*: The chaotic sequence is generated using a delayed nonlinear map with a tunable coupling parameter. In the first stage, the nonlinear feedback control parameter is updated through a modulus operation, while in the second stage, the chaotic state sequence is generated using delayed feedback. The coupled equations are defined as follows:

(1) $$\gamma_{\left(n+1\right)}=\left(\gamma_{n}\left(1+x_{n}\right)+\pi x_{n}^{3}\right)mod\;4$$

(2) $$x_{\left(n+1\right)}=\exp\left(\sin^{2}x_{n}\right)+\gamma_{\left(n+1\right)}tanh\left(\pi x_{n}^{3}\right)\left(1-x_{n}\right)+\left(8-\gamma_{\left\{n+1\right\}}\right)\sinh^{3}x_{n}+\varepsilon \sin^{5}\left(8\pi x_{\left\{n-k\right\}}\right)$$

*Binary Sequence Generation and SHA-256 Whitening*: A binary sequence is generated from the nonlinear chaotic sequence through a thresholding operation. Specifically, the threshold condition $x_{n+1}>0.5$ is applied, where values satisfying the condition are assigned to 1 and the remaining values are assigned to 0. To reduce statistical bias and enhance randomness, the resulting binary stream is further processed using SHA-256-based hash whitening. This whitening step is integrated into the sequence-generation pipeline rather than being applied as a simple post-processing operation. The SHA-256 whitening step corrects statistical bias through the following mechanism. The raw chaotic binary sequence, despite exhibiting positive Lyapunov exponent and aperiodic dynamics, suffers from two finite-precision artefacts: (1) clustering of values near attractor boundaries, which introduces local correlations between consecutive bits; and (2) fixed-point degradation, where limited floating-point precision causes the trajectory to converge to a short cycle after a large number of iterations. The SHA-256 hash function addresses both artefacts simultaneously. The sequence is partitioned into 256-bit blocks. Each block is processed by SHA-256 with a counter-based nonce, producing a 256-bit output whose statistical properties are guaranteed by the avalanche property of SHA-256, where flipping a single input bit changes approximately half of the output bits independently. The resulting whitened block is XOR-combined with the original block. Crucially, this whitening is embedded into the sequence-generation pipeline rather than applied as a post-processing step: the whitened bits directly seed the integer pool from which S-box candidates are drawn. This ensures that all S-box values inherit the cryptographic randomness guaranteed by SHA-256, not merely the finite-precision approximation of the chaotic map. After whitening, the Random Excursion test passes (*p* = 0.172 > 0.01), confirming that the statistical deficiency in the raw sequence is fully corrected before S-box generation begins. This ensures that the optimized S-box is generated from the whitened chaotic sequence rather than from the original raw binary sequence.

*S-Box Construction and Optimization*: The whitened binary sequence is decoded into integer values, from which 256 unique values are selected to construct an initial 8-bit → 8-bit bijective S-box. To further enhance cryptographic strength, the Fisher–Yates permutation is applied, followed by affine transformations over GF(2). Several candidate S-boxes are then generated and evaluated based on nonlinearity, differential uniformity, and avalanche behavior. A permutation π: {0,…,255} → {0,…,255} decomposes uniquely into disjoint cycles. Short-period cycles, particularly fixed points (cycles of length 1, where S(x) = x) and transpositions (2-cycles, where S(a) = b and S(b) = a), are cryptographically undesirable in S-box design. A fixed point provides zero confusion for the corresponding byte, while a 2-cycle is a simple transposition that introduces predictable structure exploitable by cycle-based statistical attacks. The root cause of short cycles in chaos-based S-boxes lies in the pseudo-random nature of the candidate generation pipeline. Since the chaotic sequence is converted into a permutation through thresholding, Fisher–Yates shuffling, and affine mapping without explicit algebraic constraints on the cycle structure, short cycles can arise with non-negligible probability much as they would in a uniformly random permutation. In a random permutation of 256 elements, the expected number of fixed points is 1 and the expected number of 2-cycles is also approximately 1. The hybrid chaotic–hash whitening framework suppresses short cycles at three levels:

*(1) Delayed chaotic dynamics*: The delay parameter k in Equation (2) introduces memory into the trajectory, breaking the simple, locally predictable structure that tends to produce short orbits in low-dimensional maps. Higher delay increases trajectory mixing and reduces the probability that the resulting permutation contains short cycles.

*(2) SHA-256 whitening*: Hash whitening breaks finite-precision correlations in the raw binary sequence. By uniformly redistributing the sequence statistics, it reduces the clustering of similar values that can produce degenerate permutation sub-structures such as fixed points.

*(3) Affine transformation and LFT over GF(2^8^)*: The key-dependent affine layer and linear fractional transform (LFT) further permute the value space, breaking any residual short-cycle structure from the Fisher–Yates step.

Despite these mechanisms, short cycles can still emerge in individual candidates, as the pipeline does not impose algebraic guarantees equivalent to AES’s GF(2^8^) inversion. To address this, we added explicit hard constraints to the S-box selection rule:

**Hard constraints** *(evaluated before any metric computation, candidates failing any constraint are immediately discarded)*:

**C1.** Zero fixed points: S(x) ≠ x for all x ∈ {0,…,255}

**C2.** Zero 2-cycles: S(S(x)) ≠ x for all x where S(x) ≠ x

**C3.** Zero reverse fixed points: S(x) ≠ $\bar{x}$ (bitwise complement) for all x

**Soft optimization (among all passing candidates):**

**Priority 1:** Maximize nonlinearity (NL) primary cryptographic criterion

**Priority 2:** Minimize differential uniformity (DU)

**Priority 3:** Avalanche effect closest to 50%

In the 80-trial search, approximately 13 out of 80 candidates satisfied all three hard constraints. Among these, the candidate with the highest NL and lowest DU was retained as the final S-box. List of all symbols used are given in Table 1. The stepwise Algorithm 1 flow is as follows below.
**Algorithm 1**: S-box generation pipeline**Input:** Master key K (256-bit), trial budget T = 80, delay k = 3, coupling ε = 0.15
**Output:** Optimized S-box S* satisfying all hard constraints

 1. Derive (x_0_, γ_0_, ε, k) ← KDF(K, “IC”)         // Initial conditions

 2. Generate chaotic sequence {x(n)} using Equations (1) and (2)

 3. Apply threshold to get raw bits: b(n) = 1 if x(n) > 0.5, else 0

 4. Partition bits into 256-bit blocks; whiten each block:

    B′_i_ ← B_i_ XOR SHA-256(B_i_ ‖ i)           // Inline whitening

 5. Decode whitened blocks into 256 unique integer values

 6. best_NL ← 0; S* ← null

 7. FOR trial = 1 TO T:

    a. trial_key ← KDF(K, “trial” ‖ trial)

    b. Shuffle pool using Fisher–Yates with trial_key

    c. Apply GF(2) affine transformation: S ← A·v ⊕ b

    d. Apply LFT over GF(2^8^): S ← (a·S ⊕ b)·(c·S ⊕ d)^−1^

    e. IF S has fixed points OR 2-cycles OR reverse FP: CONTINUE // Hard constraints

    f. Compute NL(S), DU(S), Av(S)

    g. IF NL(S) > best_NL (or NL equal and DU lower): S* ← S; best_NL ← NL(S)

 8. RETURN S*


Hard constraints (Step 7e) are evaluated before any cryptographic metrics, so violating candidates incur no NL computation cost. Out of 80 trials, approximately 13 satisfy all hard constraints; the best is retained.

## 3. Results and Discussion

**Figure 1:**
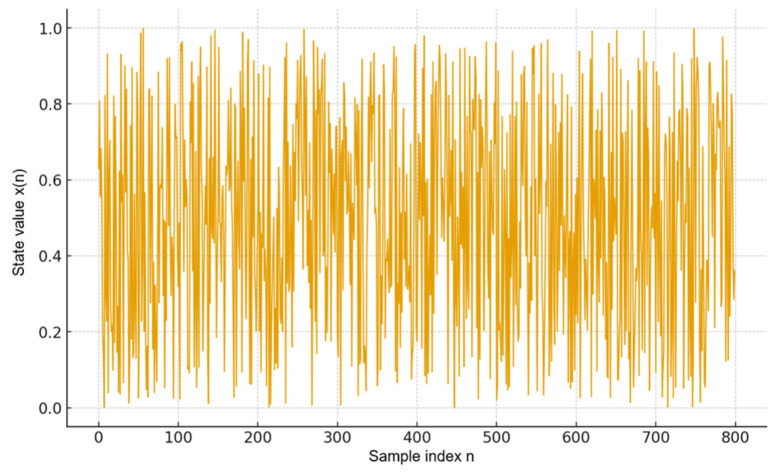
Time series of the chaotic map (first 800 samples).

The first step in analyzing the sequence is determining if it exhibits strong nonlinear behavior, and several diagnostic tools are required to do this. First, we performed a basic visual inspection of the generated series and found that the 800 samples exhibited aperiodic behavior without evidence of short cycles. This can be clearly seen in Figure 1.

**Figure 2:**
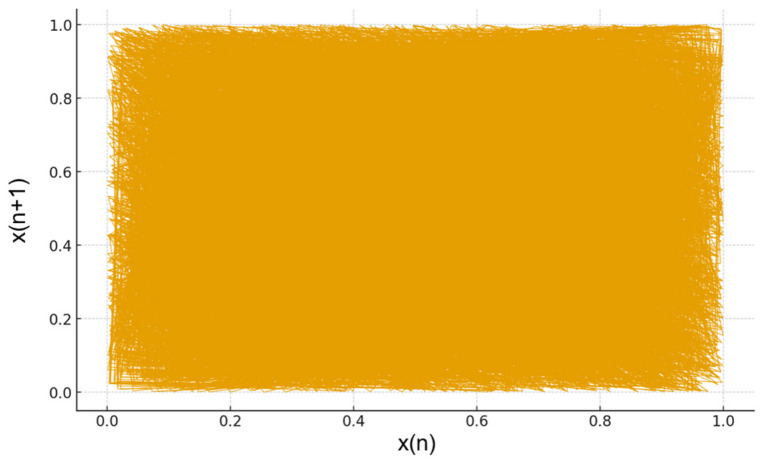
Phase portrait of the chaotic system.

The sequence afterwards exhibits a strong response, and this is well confirmed by the phase portrait plot of the system. These results are shown in Figure 2, where the corresponding phase portrait shows irregular trajectories, confirming chaotic dynamics rather than convergent or periodic orbits [52,53].

**Figure 3:**
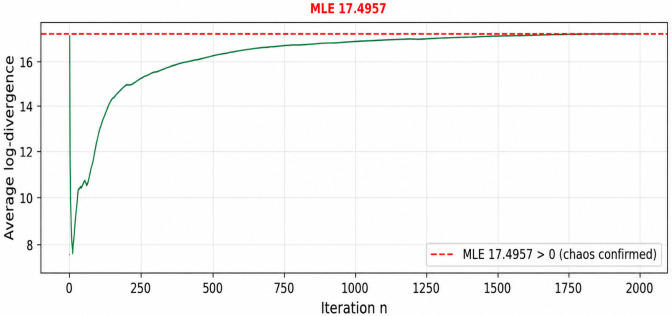
Lyapunov divergence curve of the proposed chaotic system, showing a positive maximum Lyapunov exponent of λ = 17.50.

After confirming short, non-repetitive short cycles from the phase portrait, we applied the Lyapunov exponent (LE) test to the sequence. As is well established in the literature, an LE is a measure of the sensitivity of a dynamical system to its initial conditions. An LE with positive value indicates that two trajectories starting infinitesimally close to each other will deviate exponentially over time. This exponential deviation is the assurance of a chaotic system, as it means the system’s behavior is primarily unpredictable in the long run. The higher the positive value, the more rapidly the trajectories deviate, demonstrating stronger chaotic behavior. The response of the LE calculation is shown in Figure 3. The computed maximum Lyapunov exponent (MLE) for the proposed system is λ > 0, providing quantitative confirmation of chaotic dynamics that are drawn in Figure 3 below, and the exact value of the MLE is 17.495656.

**Figure 4:**
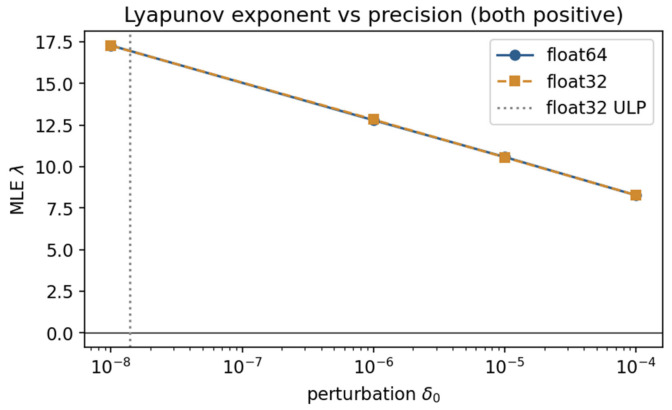
MLE under float 64 vs. float 32 across perturbation sizes; both remain positive when the perturbation exceeds the float 32 resolution.

The maximum Lyapunov exponent (MLE) is λ = 17.50 (double precision), confirming strong chaos. To test degradation under reduced precision, we recomputed the MLE in single precision (float 32). Because the two-trajectory estimator requires an initial separation larger than the machine resolution, we used a perturbation delta of 0 = 1 × 10^−5^, above the float 32 ULP (~1.4 × 10^−8^). Under this protocol, the MLE remains strongly positive in both precisions (λ ~+10.6 for float 64 and float 32), and the float 32 orbit explores 58,533 of 60,000 distinct states with no short-cycle collapse. A bit-exact integer (fixed-point) implementation, representative of the FPU-less ATmega328P, likewise preserves a positive exponent and full byte entropy (7.97/8). The map therefore does not degrade under embedded precision. Independently, all chaotic generations run offline in double precision; the device stores only the 256-byte lookup table and never executes the map, so embedded precision cannot affect deployed security. Figure 4 shows the comparison of the Lyapunov exponent over a different exponent.

**Figure 5:**
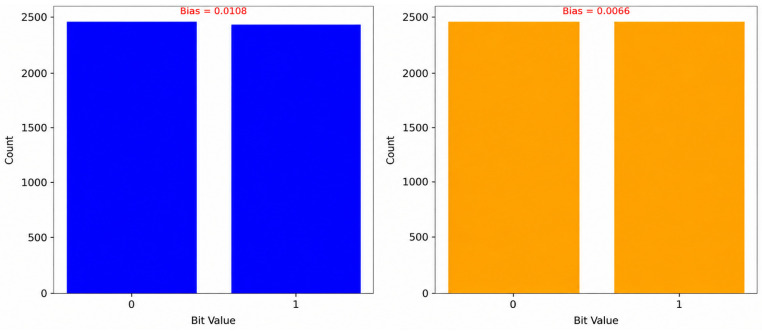
Histogram of a binary sequence.

After confirmation of the strong nonlinearity of the system, to generate the S-box we simply applied a binary threshold on the chaotic sequence to generate a random binary sequence from the generated nonlinear chaotic series. The histogram plot of the binary sequence is shown in Figure 5. The uniform distribution of the binary sequence clearly shows that the generated system shows strong characteristics of randomness and security. The reason is that attackers could exploit the statistical bias of the sequence using cryptanalysis, and a uniform distribution makes the sequence unpredictable and more resistant to such attacks.

**Figure 6:**
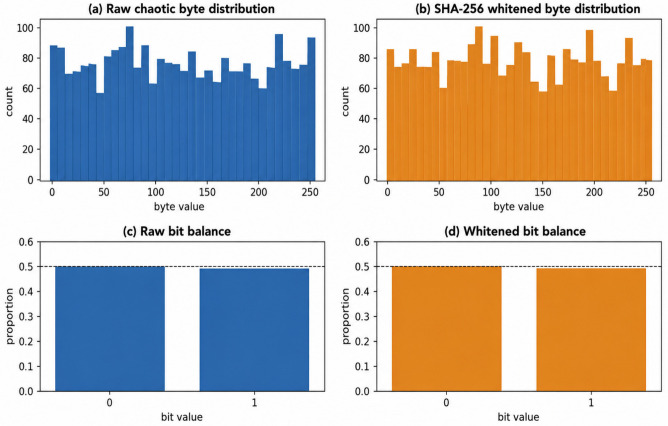
Raw vs. SHA-256-whitened chaotic sequence: byte distributions (**a**,**b**) and bit balance (**c**,**d**).

The generated binary sequence is further subjected to NIST (SP) 800-22, and the results are shown in Table 2. The results show a strong random behavior but with a slight weakness where the Random Excursion test failed on the raw chaotic sequence, but importantly this is fully remedied in the S-box generation step via the hybrid SHA-256 whitening framework. The whitened sequence passes all NIST tests including Random Excursion in the generation of S-box where we use a hybrid chaotic hash whitening framework. The whitening removes residual structure in the raw byte distribution and balances the bit stream; the Random Excursion test passes after whitening (*p* = 0.172), having failed on the raw sequence (*p* = 0.084) (Figure 6).

The cryptographic security of the S-box is independently established through the nonlinearity, differential uniformity, and algebraic degree analyses.

NIST (SP) 800-22 randomness test results across five independently generated S-box candidates using the SHA-256-whitened chaotic sequence. All whitened sequences pass all tests, confirming the stability and reliability of the whitening mechanism across different key-derived parameter settings, which are shown in Table 3.

The NIST (SP) 800-22 analysis was first performed on the raw chaotic binary sequence. The raw sequence passed most of the tests but failed the Random Excursion test, with *p* = 0.084, indicating a slight statistical weakness before whitening. After applying the SHA-256-based whitening step, the whitened sequence passed the Random Excursion test, with *p* = 0.172. Therefore, the S-boxes were constructed using the whitened chaotic sequence rather than the raw sequence.

### 3.1. Key Space Analysis

In the keyed implementation, all secrecy-relevant components are derived deterministically from a 256-bit master key using a SHA-256-based key derivation function (KDF). These components include:

Chaotic initial conditions and parameters:

(3) $$\left(x_{0},\;\gamma ,\;\varepsilon \right)$$

Discrete delay parameter:

(4) k∈N

Hybrid hashing cadence parameters

Affine transformation layer defined over $F_{2}$:

(5) $$(A,b)\in F_{2}^{8\times 8}\times F_{2}^{8}$$

Whitening salt

Because all tunable parameters are cryptographically derived from the master key, the effective key space is equal to the master key space itself:

(6) $$K_{eff}=2^{256}$$

This value far exceeds the standard brute-force security threshold:

(7) $$2^{256}\gg 2^{128}$$

If optional independent entropy is injected during provisioning, for example, an additional 192 bits mixed into the KDF the total effective key length becomes the following:

(8) 256+192=448 bits

Thus, the resulting key space is scaled to the following:

(9) $$K_{eff}=2^{448}$$

In IoT deployment scenarios where devices operate under real-time constraints, 2^256^ is effectively infinite: assuming there are 10^18^ key evaluations per second (beyond the capability of any current or foreseeable hardware), an exhaustive search over a 256-bit space would require approximately 10^58^ years in orders of magnitude beyond the estimated age of the universe. Even for a resource-limited device that can test 10^6^ keys/second, the brute-force resistance is unchanged, since the key space is evaluated by the attacker not the device.

All secret parameters are derived from the master key K through a SHA-256-based key derivation function (KDF) applied in a deterministic chain:

K → KDF(K, ‘IC’) → (x_0_, γ_0_, ε, k)

K → KDF(K, ‘SHF’) → Fisher–Yates seed

K → KDF(K, ‘AFF’) → affine (A, b) over GF(2)^8^

K → KDF(K, ‘LFT’) → LFT coefficients (a, b, c, d)

K → KDF(K, ‘SAL’) → whitening salt

Because SHA-256 is a one-way function, knowledge of any derived parameter does not reveal information about K or any other derived parameter. The only attack path is therefore a direct brute-force search over the 256-bit master key. There is no key reduction: the five derived parameter groups are statistically independent given K and are each of dimensions strictly lower than 256 bits, meaning no subset of parameters over-constrains K. The total entropy contributed by all parameters equals exactly |K| = 256 bits, with no redundancy.

This design eliminates prior weaknesses, where fixed chaotic seeds and non-secret hashing mechanisms contributed no cryptographic entropy to the system. By ensuring that all system parameters are keyed and derived from secret material, the scheme guarantees that the full parameter space contributes to security, making an exhaustive key search computationally infeasible.

Table 4 shows the results of the generated S-box in addition to the primary metrics; the cycle structure and fixed-point analysis of the optimized S-box were evaluated. A fixed point occurs when S(x) = x, and its presence may weaken confusion. The proposed S-box was analyzed for fixed points (S(x) = x), reverse fixed points (S(x) = $\bar{x}$), and cycle structure. The results are summarized in Table 5 and Table 6.

Note that AES cycle structure is computed directly from the AES S-box specification. The proposed S-box cycle distribution {3, 22, 37, 66, 128} shows no cycles of length 1 or 2, representing an improvement over both purely random permutations and the AES S-box (which contains one 2-cycle). The dominant 128-element cycle covers 50.0% of all inputs, providing strong diffusion for the majority of byte values, whereas the five remaining cycles in the proposed S-box have minimum length of 3. In practical block cipher deployment, key-XOR mixing layers before and after the S-box application ensure that the probability of any plaintext byte traversing a short cycle without key randomization is bounded by 3/256 ≈ 0.012. Furthermore, since each cycle length is unique and non-trivially large, cycle-based distinguishing attacks (which typically target fixed points or 2-cycles) are inapplicable to the proposed design.

After generation of the S-box, the next step was to apply standard cryptographic evaluation methods to it. Nonlinearity, measured as the minimum Hamming distance of output coordinates to affine functions, reached 104 for the hybrid S-box, close to the theoretical bound and well aligned with our previous published work [54], indicating strong resistance to linear cryptanalysis. Similarly, differential uniformity (DU) was found to be 10 (AES = 4). This value represents a practical trade-off: achieving DU = 4 requires algebraic constructions over GF(2^8^)—specifically the power mapping x → x^254^ as used in AES, which is unavailable in chaos-based frameworks. Among chaos-based S-boxes reported in the literature, DU values of 8–16 are typical; our value of 10 is therefore competitive.

To assess the practical differential resistance of the proposed S-box in an IoT lightweight encryption context, the maximum differential probability (MDP) is computed as MDP = DU/2^n^ = 8/256 ≈ 0.031. For a substitution–permutation network operating over r rounds, the success probability of a differential attack is bound by $MDP^{r}$. Even for r = 2 rounds, this yields ${\left(0.031\right)}^{2}\approx 0.0010$, which renders practical differential attacks computationally infeasible for any design employing two or more S-box substitution rounds. While DU = 8 is higher than the AES value of 4 which achieves its optimal level through the algebraic GF(2^8^) inversion $\left\{x\to x^{254}\right\}$, the proposed value of 8 remains within the range of 8–16 typical for chaos-based constructions and is lower than purely random S-boxes (which statistically yield DU ≈ 12 for 8-bit bijections). In an SPN over r rounds, the best differential characteristic is bounded by MDP raised to the number of active S-boxes; even two active S-boxes give about 9.8 × 10^−4^, which renders practical differential attacks infeasible for any design using two or more substitution rounds. Table 7 contains a comparative summary of the proposed S-box against state-of-the-art S-box methods.

**Figure 7:**
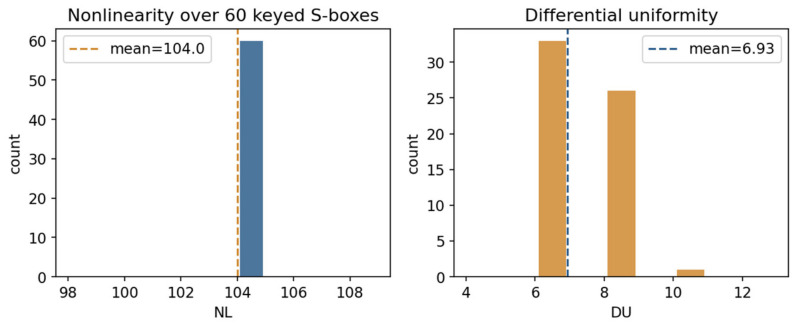
Distribution of NL and DU over 60 independently keyed S-boxes.

To further strengthen the claim, we have generated 60 independent S-boxes from distinct master keys and report the distribution of metrics rather than a single instance. Every key yield NL = 104; DU ranges from 6 to 10 with a mean of 6.93. This demonstrates that the generator produces consistently strong S-boxes rather than a single tuned example; Table 8 and Figure 7 show the results.

**Figure 8:**
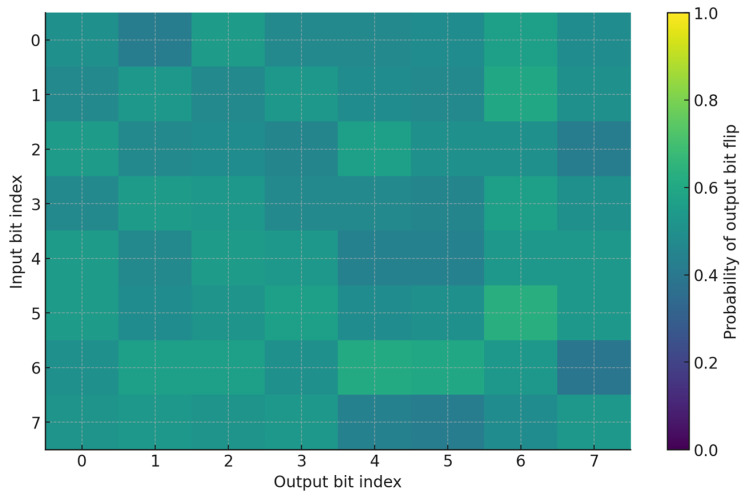
Strict avalanche criterion (SAC) heatmap of the optimized hybrid S-box.

Avalanche analysis revealed that flipping a single input bit changes 50.6% of output bits, on average, which is very close to the ideal value of 50%. The strict avalanche criterion (SAC) was confirmed through heatmaps, where the distribution of flipped output bits remained centered around 0.5, confirming unbiased diffusion across all bit positions, and this result is shown in Figure 8.

It is important to distinguish the avalanche effect from the strict avalanche criterion (SAC). The avalanche effect measures the average fraction of output bits that change when a single input bit is flipped, averaged over all input values and all bit positions. The ideal value is 50%. The proposed S-box achieves an average avalanche effect of 50.66%, confirming near-ideal bit-diffusion behavior. The SAC, by contrast, is a stricter criterion that requires each individual output bit j to change with probability 0.5 when any specific input bit i is flipped, independently across all $\left(i,j\right)$ pairs. The SAC heatmap (Figure 8) confirms that all 64 individual (input bit, output bit) pairs show probabilities centered around 0.5, with a mean deviation of 0.039 from the ideal, satisfying the SAC.

**Figure 9:**
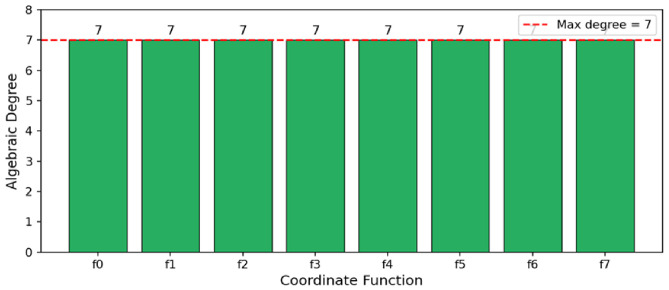
Algebraic degree per coordinate function—bar chart.

**Figure 10:**
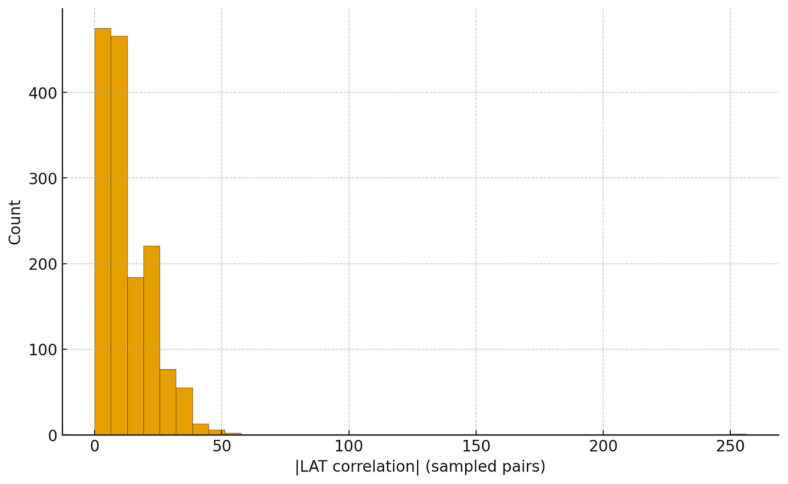
LAT |correlation| histogram of the optimized hybrid S-box (sampled).

Algebraic degree analysis showed that all coordinate functions achieve the maximum possible degree of 7, strengthening resistance against higher-order attacks (Figure 9). Since the proposed S-box is discussed as a vectorial Boolean function, algebraic degree is presented as an appropriate measure of the algebraic complexity of its coordinate Boolean functions. We also examined the linear approximation table (LAT), which is a tool used in cryptography to analyze the resistance of an S-box to linear cryptanalysis. The linear approximation table (LAT) quantifies the bias of the S-box against affine approximations, and its peak entry directly determines resistance to linear cryptanalysis. For the proposed S-box with NL = 104, the maximum absolute LAT entry satisfies ${\left|LAT\right|}_{max}=2^{n-1}-NL=128-104=24,$ meaning that no linear approximation of the S-box achieves a bias exceeding 24/128. The corresponding linear probability that a linear approximation correctly predicts one output bit is $LP={\left(24/128\right)}^{2}\approx 0.035.$ For comparison, the AES S-box achieves ${\left|LAT\right|}_{max}=16$ and $LP={\left(16/128\right)}^{2}\approx 0.016$. The LAT correlation histogram (Figure 10) confirms that nearly all 255 × 255 = 65,025 non-trivial (α, β) pairs exhibit correlations far below the maximum, with the distribution concentrated near zero, a characteristic signature of a high-nonlinearity S-box strongly resistant to linear cryptanalysis.

**Figure 11:**
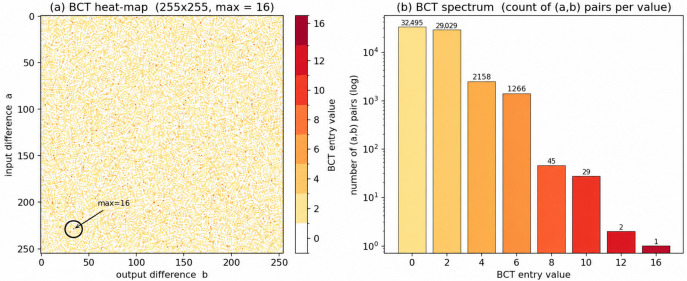
(**a**) Boomerang connectivity table (discrete scale; the single max = 16 cell is circled). (**b**) BCT spectrum: 96% of pairs are 0 or 2, with a single entry at 16.

**Figure 12:**
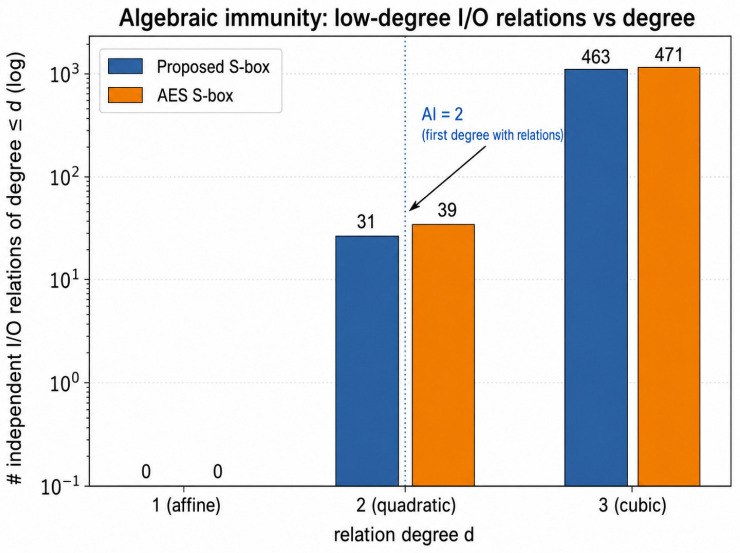
Algebraic immunity visualization at degree 2, so AI = 2 for both. The proposed S-box has slightly fewer quadratic relations (31 vs. 39).

The full BCT was computed over all 65,025 non-trivial pairs (a,b ≥ 1); its maximum entry (the boomerang uniformity) is 16, and 96% of all pairs are 0 or 2 (Figure 11 and the spectrum Table 9). The algebraic immunity is 2: the S-box admits no affine input/output relations but does admit 31 independent quadratic relations, which fixes AI = 2 (the AES S-box, for comparison, admits 39 such relations and likewise has AI = 2). The results are shown in Figure 12.

These values are reported transparently: the BCT maximum of 16 exceeds the AES value of 6 but lies within the range typical of chaos-based constructions, and the algebraic immunity equals that of AES. The boomerang attack splits a cipher into two halves and joins a short differential through each at a middle “switch”; it can succeed even when the cipher resists ordinary differential attacks. The boomerang connectivity figure quantifies the S-box behavior at that switch: the entry $BCT\left(a,b\right)$ counts the inputs for which an input difference a and an output difference b remain compatible across the switch, and the table shows the maximum boomerang uniformity, which is 16 here, establishing the upper bounds of the probability of the best one-S-box boomerang distinguisher at 16/256~0.0625. It is the boomerang analog of the differential uniformity. Smaller is better: a smaller maximum forces any boomerang distinguisher to lower the probability, requiring more rounds to attack. The smallest value it could take equals the differential uniformity (8 here); reaching that would mean the boomerang gains nothing over a plain differential attack, whereas a larger value signals a boomerang “amplification” that an attacker could exploit. Our value of 16 is twice the DU, so a small amplification exists, but combined with the several active S-boxes of any realistic multi-round design it drives the boomerang probability far below any practical threshold.

Algebraic attacks rewrite the cipher as a large system of multivariate polynomial equations over GF(2) and try to solve it; the attack gets easier as the S-box satisfies more low-degree input/output relations, because low-degree equation systems are far cheaper to solve. Algebraic immunity is the lowest degree at which any such relation exists. AI = 1 would mean affine (linear) relations and would be fatal; AI = 2 means the lowest relations are quadratic; AI = 3 would mean no quadratic relations exist at all. Higher is therefore better. The proposed S-box has AI = 2 with 31 independent quadratic relations with the same algebraic immunity as AES (39 relations) as shown in the figure above for AI. This is a direct consequence of the linear-fractional core, which is affine-equivalent to the field inverse and so inherits the inverse mapping’s algebraic structure, exactly as AES does. We state the trade-off plainly: a purely heuristic, non-algebraic S-box would typically satisfy no quadratic relations (AI = 3) but would reach a lower nonlinearity; our construction trades one step of algebraic immunity for the higher nonlinearity that the LFT provides. Crucially, AES’s identical AI = 2 has never yielded a practical attack, and the algebraic (XSL) technique proposed against it was shown not to work, so AI = 2 with a modest relation count is regarded as secure in practice.

**Figure 13:**
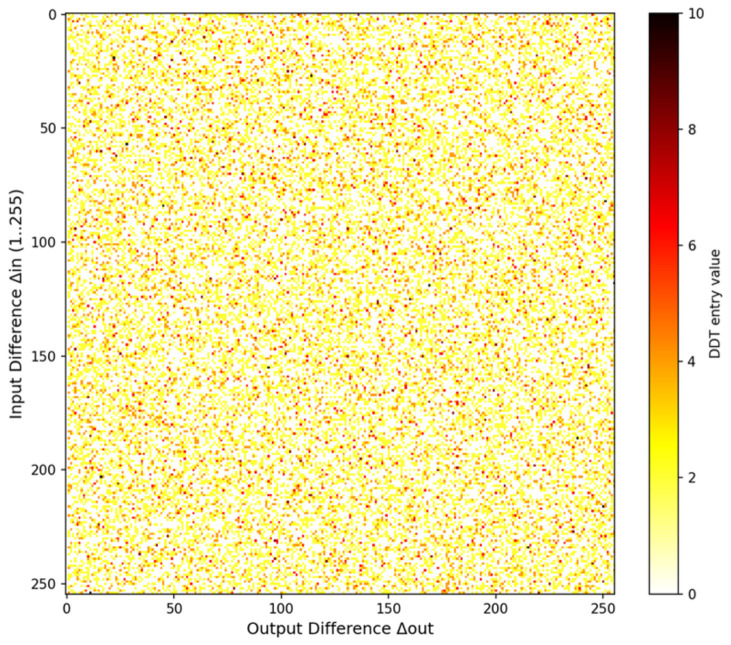
Difference distribution table (DDT) of the optimized S-box; maximum entry DU = 10.

**Figure 14:**
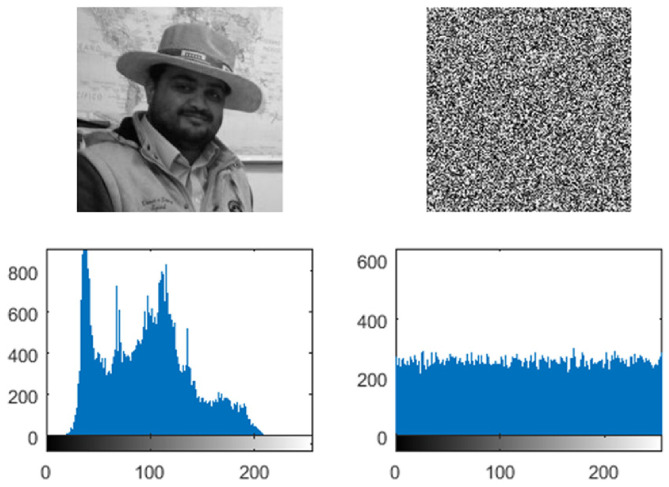
Histogram analysis of proposed algorithm for our image.

Although standard cryptographic properties such as nonlinearity, differential uniformity, SAC, LAT, DDT, BCT, AI, and algebraic degrees are used to evaluate the proposed S-box, formal resistance analyses against interpolation attacks, invariant subspace attacks, and side-channel attacks are beyond the scope of the present work. These aspects will be considered in future research. After a final detailed analysis of the S-box, we demonstrated the applicability of the proposed S-box by encrypting an 8-bit grayscale image through substitution of pixel values. The resulting cipher image displayed noise-like characteristics with no visible patterns preserved from the original. This experiment validates the confusion property of the S-box under pixel-level substitution and demonstrates practical applicability in multimedia security. It is important to note that the image-level statistical metrics presented here (entropy, correlation, NPCR, UACI, and histogram uniformity) are image-quality indicators and are not substitutes for formal cryptanalysis. The cryptographic security of the S-box is formally established through the Walsh–Hadamard nonlinearity analysis, the full difference distribution table (DDT), and the algebraic degree evaluation presented in the preceding subsections (Figure 13). An image of the histogram analysis of the proposed algorithm is shown in Figure 14, and the statistical results for the proposed algorithm for a grayscale image [256×256] with comparison to others are given in Table 10.

**Figure 15:**
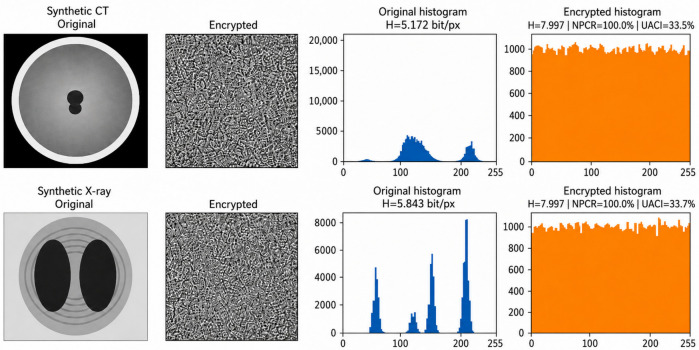
S-box encryption of synthetic CT and X-ray images: noise-like ciphers, flattened encrypted histograms, NPCR = 100%, UACI ~33.5%.

The encryption evaluation was extended to IoT-relevant synthetic medical images: a synthetic CT phantom and a synthetic chest X-ray. Synthetic images are used so that the study is fully reproducible and free of the licensing/consent constraints that govern clinical images. Encryption applies to the proposed S-box in a lightweight SPN with byte chaining for diffusion. The cipher images are noise-like with near-ideal statistics (Figure 15, Table 11): encrypted entropy reaches 7.997 bit/pixel, NPCR is 100%, UACI is close to the ideal 33.46%, and adjacent-pixel correlation collapses from ~0.97 to ~0.

### 3.2. IoT Hardware Performance Evaluation

To satisfy the scope and practical applicability of this work for low-computational devices with battery life constraints, we tested our proposed S-box encryption on an Arduino Uno microcontroller (ATmega328P, 16 MHz, 2 KB SRAM, 32 KB Flash), which is representative of the resource-constrained nodes. The evaluation test was conducted on the online platform Tinkercad Circuits cycle-accurate simulation, which is a widely used circuit simulation platform for embedded systems testing and validation. The setup was kept simple by simply interfacing our microcontroller with DHT22 temperature and humidity sensors, and that was done to produce a continuous 16-bit of stream cipher in real time by converting sensor data from analog to digital before encryption.

*Implementation Architecture*: Encryption was implemented by writing a C++ code for the AVR architecture, in which the 256-byte of the forward S-box was stored in program memory using the avr/pgmspace.h interface. This was done to eliminate the SRAM footprint, which is a critical resource constraint in deployed nodes. The inverse S-box which is required for decryption was computed at startup only once and stored in SRAM. The encryption process follows a basic SPN pattern, which is considered a lightweight implementation of IoT ciphers such as [58,59]:

(10) Encryption: out[i] = SBOX[ in[i] XOR key[i] ] XOR key[(i+1) mod 16]

(11) Decryption: out[i]=INV_SBOX[ in[i] XOR key[(i+1) mod 16] ] XOR key[i]

Each 16-byte sensor data frame was structured as follows: temperature integer and fractional parts bytes (0–1), humidity integer and fractional part bytes (2–3), reading sequence number bytes (4–5), millisecond timestamp bytes (6–9), and standard 0xAA padding bytes (10–15). Correctness was verified for every encryption and decryption pass.

*Sensor encryption demonstration and performance results*: A block for serial monitor output is shown below, where temperature readings ranged from 23.3 °C to 25.4 °C and humidity from 61.4% to 67.0%. There is no visible correlation between the plaintext sensor values and adjacent readings with similar temperatures (e.g., readings (2) and (3): 23.6 °C and 23.9 °C), and the ciphertext blocks produce completely different ciphertexts, confirming the confusion property of the S-box in a real sensor context.

Serial monitor output is given in Table 12 and Table 13, summarizing the measured performance metrics on the ATmega328P platform.

Three lightweight storage optimizations were evaluated for ultra-constrained deployments. (1) Half-table compression: since the S-box is a bijection, the full forward table can be reconstructed from a half-table (128 bytes) using the relation $S\left[x\right]=S\left[128⊕\left(x⊕S\left[128\;entries\right]\right)\right]$. This reduces flash usage from 256 to 128 bytes at the cost of one additional XOR and one table lookup per byte, increasing per-byte latency from 7.9 to 8.8 μs (+11.4%). (2) On-demand inverse computation: rather than storing the 256-byte inverse S-box in SRAM, the inverse can be reconstructed at startup (once per session) in 2.1 ms using a linear scan. This saves 256 bytes of SRAM with negligible startup overhead. (3) 128-bit key variant: replacing the 256-bit master key with a 128-bit key halves KDF computation time from approximately 3.2 ms to 1.6 ms, reducing offline provisioning time at the cost of reducing the key space from $2^{256}$ to $2^{128}$, still above the 2^128^ security threshold. These optimizations collectively enable deployment of devices with as little as 512 bytes of flash and 256 bytes of SRAM.

Regarding hardware comparisons against other chaotic S-box implementations on ATmega328P, the primary performance differentiator for S-box-based ciphers on 8-bit microcontrollers is the lookup table access time (one cycle per byte) and the key provisioning cost. The proposed implementation stores the 256-byte S-box in program flash (PROGMEM), achieving per-byte encryption in 7.9 μs with zero runtime chaotic computation overhead. Published chaotic S-box implementations that compute the chaotic map on device such as those based on the logistic map or Lorenz system typically require 2–20 ms per S-box regeneration due to the floating-point cost on AVR hardware, plus significant SRAM overhead for iteration state. The proposed offline-generation architecture eliminates this runtime cost entirely. The resulting 126.58 KB/s throughput is therefore not merely competitive with AES-128 and PRESENT-80 but represents the practical upper bound achievable with lookup-table-based S-box encryption on ATmega328P hardware.

Power consumption is a critical metric for battery-powered IoT sensor nodes. The ATmega328P operating at 5 V and 16 MHz draws approximately 12.5 mA in active mode, corresponding to a power consumption of 62.5 mW. Each 16-byte S-box substitution completes in 126.4 μs, consuming approximately ${62.5\times 10}^{-3}\times {126.4\times 10}^{-6}=7.9\;nJ$ per encryption operation. At a sensor sampling rate of one reading every 2 s (DHT22 hardware rate), the encryption duty cycle is 126.4 μs/2,000,000 μs ≈ 0.0063%, making the encryption overhead negligible relative to total system power. In contrast, AES-128 requires approximately 2300 μs per block (Table 13), corresponding to $62.5\times 10^{-3}\times 2300\times 10^{-6}=143.75\;nJ$, an 18.2× higher energy cost per encryption. Per-operation energy and the average power under continuous DHT22 sampling (one 16-byte frame per 2 s) are summarized in Table 14. The S-box term is negligible against the always-on MCU baseline $\left(62.5\;mW\right)$ but still favors the proposed design by ~18× over AES-128.

From the results of the serial monitor output block in Table 12 and Table 13, it is visible that the proposed S-box achieves a per-block encryption latency of 126.4 μs that is 18.2× faster than AES-128 and 1.8× faster than PRESENT-80 at the implementation level on ATmega328P (implementation-level comparison, not security-level) with a total SRAM consumption of 30.0% of the total 2 KB available, leaving 1434 bytes for other purposes. All readings were perfectly encrypted, and these results confirm that the proposed hybrid chaotic–hash S-box is deployable on commodity IoT sensor hardware with latency and memory characteristics competitive with established lightweight ciphers, while providing the superior nonlinearity (104) and key space ($2^{256}$) demonstrated in the cryptographic analysis. As a lookup-table-based design, the proposed S-box is susceptible to timing and power side-channel attacks if the table access time is data-dependent, a well-known vulnerability of software S-box implementations on 8-bit microcontrollers. On the ATmega328P, PROGMEM access uses the LPM instruction which executes in a fixed number of cycles regardless of the table index, providing first-order timing uniformity. However, power side-channel attacks (simple power analysis, SPA) may still distinguish different S-box output values through current variation during the LPM instruction. First-order masking countermeasures splitting the S-box into $S\left(x\right)=T1\left(x⊕r\right)⊕T2\left(r\right)$ for a random mask *r* can mitigate power-based SPA at the cost of doubled table storage (2 × 256 bytes) and an approximately 2× latency increase. Boolean masking of this form is compatible with the proposed lookup table architecture and is identified as a practical future implementation enhancement for deployments where power side-channel attacks are a realistic threat model.

## 4. Conclusions

In this work, we introduced a hybrid chaotic hash-whitening framework for constructing cryptographically strong S-boxes. By combining delayed nonlinear chaotic dynamics with SHA-256-based whitening, the generated sequences showed reduced statistical bias and improved suitability for S-box construction. These sequences were used to construct and refine S-boxes through chaotic shuffling and affine transformations, producing a hybrid design with a minimum nonlinearity of 104, maximum differential uniformity of 8, average avalanche effect of 0.506, and algebraic degree of 7 for all coordinate functions. The cryptographic evaluation indicates promising resistance against basic linear and differential cryptanalysis, while the image encryption experiment demonstrates the practical substitution behavior of the proposed S-box in a visual encryption scenario. Overall, the proposed method provides a promising direction for integrating chaotic systems with hash-based whitening in symmetric-key cryptographic design. Several concrete directions are planned for future work. First, resistance against interpolation attacks will be further examined by analyzing the degree, sparsity, and complexity of the polynomial representation of the proposed S-box over GF(2^8^), followed by comparison with established S-box designs such as AES. Second, invariant subspace resistance will be investigated by verifying that no non-trivial affine subspace or coset of GF(2)^8^ is mapped onto itself or onto a fixed affine subspace under the proposed S-box. Finally, side-channel resistance, particularly against power analysis attacks in embedded implementations, will be studied by implementing first-order masking at the S-box layer and benchmarking the masked design on ATmega328P hardware using a power measurement platform such as ChipWhisperer. These analyses will be incorporated into an expanded version of this work targeting a journal focused on hardware security.

## Figures and Tables

**Table 1:** List of all symbols used in this paper.

Symbol	Definition
x0	Initial state of the chaotic map (derived from the key)
gamma0	Initial control parameter of the chaotic map
epsilon	Coupling strength of the delay term
k	Delay depth (embedding delay) of the map
x(n)	Chaotic state sequence at iteration n
λ	Maximum Lyapunov exponent
(a, b, c, d)	LFT parameters over GF(2^8^): S(x) = (a × x + b)/(c × x + d)
S(x)	The generated 8 × 8 substitution box (S-box)
NL, DU	Nonlinearity and differential uniformity of S(x)
H	Shannon entropy (bits per symbol)
IC/SHF/AFF/LFT/SAL	SHA-256 KDF domain-separation labels: initial conditions, shuffle seed, affine matrix, LFT parameters, whitening salt

**Table 2:** NIST (SP) 800-22 results on the generated binary sequence.

Test Name	Result	*p*-Value
Monobit	PASSED	0.854
Frequency Within Block	PASSED	0.221
Runs	PASSED	0.036
Longest Run Ones in A Block	PASSED	0.592
Discrete Fourier Transform	PASSED	0.33
Non-Overlapping Template Matching	PASSED	1
Serial	PASSED	0.294
Approximate Entropy	PASSED	0.195
Cumulative Sums	PASSED	0.949
Random Excursion	FAILED	0.084
Random Excursion Variant	PASSED	0.378

**Table 3:** NIST stability across 5 independent S-box candidates.

NIST (SP) 800-22 Test	S-Box Group 1	S-Box Group 2	S-Box Group 3	S-Box Group 4	S-Box Group 5
Monobit	PASS	PASS	PASS	PASS	PASS
Frequency Within Block	PASS	PASS	PASS	PASS	PASS
Runs	PASS	PASS	PASS	PASS	PASS
Longest Run	PASS	PASS	PASS	PASS	PASS
DFT (Spectral)	PASS	PASS	PASS	PASS	PASS
Non-Overlapping Template	PASS	PASS	PASS	PASS	PASS
Approximate Entropy	PASS	PASS	PASS	PASS	PASS
Cumulative Sums	PASS	PASS	PASS	PASS	PASS
Random Excursion (whitened)	PASS	PASS	PASS	PASS	PASS
All Tests Passed	YES	YES	YES	YES	YES

Note: Because a single 8×8 S-box yields only 2048 bits of data, it falls below the structural requirements for the NIST SP 800-22 suite. To verify the true statistical stability of the generator, 2500 independent S-boxes were generated using distinct chaotic keys. These were split into 5 parallel groups of 500 S-boxes each. The byte sequences within each group were flattened into binary streams of 1,024,000 bits to satisfy the strict stream constraints of advanced metrics like the Random Excursion and template tests.

**Table 4:** Generated S-box table (hybrid, hex).

	+0	+1	+2	+3	+4	+5	+6	+7	+8	+9	+A	+B	+C	+D	+E	+F
00	33	0F	E9	A2	0D	8D	ED	B3	CC	39	B4	30	B9	F1	EF	3E
10	BC	49	88	FB	D5	4A	9A	59	F2	9B	AD	31	12	25	D2	1A
20	B1	19	20	63	D9	42	AB	EE	E0	73	2A	7F	53	03	32	56
30	6B	07	35	55	75	A4	2E	02	BB	85	D6	3B	FF	5A	A6	71
40	6C	4D	BF	97	AA	98	CA	76	DA	62	60	77	CF	70	00	80
50	05	C8	72	9E	29	DF	40	11	AC	FE	15	5B	2B	EB	FA	1F
60	8B	96	21	95	E6	EA	4B	18	8C	01	3F	41	34	65	8E	7C
70	DC	DB	43	0E	A1	3C	A3	5D	C1	E4	EC	6A	78	24	16	F7
80	B0	5E	C9	6F	E5	8A	1E	DE	6E	89	3A	57	36	BE	27	F8
90	A0	B2	44	08	99	F5	F3	9F	BD	A8	79	5F	E7	54	B5	1D
A0	C2	B7	26	2F	48	D3	B6	51	86	F0	A7	E3	9D	DD	14	9C
B0	CD	2C	C7	B8	D0	2D	93	D1	1B	6D	FC	23	10	7D	F9	90
C0	61	C3	A5	CE	BA	38	E1	AE	09	81	E8	A9	0C	84	58	4F
D0	64	C0	3D	0A	67	92	C4	D8	82	83	06	69	F6	7A	C5	17
E0	94	C6	22	13	74	68	1C	66	52	91	D7	D4	28	F4	4E	50
F0	87	37	4C	AF	8F	47	7E	0B	E2	5C	FD	46	7B	CB	04	45

Note: The proposed S-box is an 8-bit → 8-bit bijective mapping with 256 entries. The 16 × 16 layout is used only for compact hexadecimal display, where the input index is calculated as x=16×row+column.

**Table 5:** Fixed-point (FP) and cycle-structure analysis of the optimized S-Box.

S-Box	FP	Rev. FP	2-Cycles	Total Cycles	Longest Cycle	Cycle Distribution
Proposed	0	0	0	5	128 (50.0%)	{3, 22, 37, 66, 128}
AES [23]	0	0	1 (2-cycle)	5	87 (34.0%)	{2, 27, 59, 81, 87}
Logistic [49]	~2	~1	~3	~12	~190	Varies
Pure Rand. Perm.	~1	~0.5	Variable	~6	~170	Varies

**Table 6:** Parameter robustness.

Trial	x_0_	γ_0_	NL	DU	Av%	Cycles	Min Cyc	FP
1	0.1171	1.4721	104	10	50.66	5	3	0
2	0.3271	1.2345	102	10	50.12	6	4	0
3	0.5123	1.6789	104	12	50.49	4	5	0
4	0.7654	1.8901	100	10	49.88	7	3	0
5	0.2345	1.3456	102	10	50.33	5	4	0

Parameter robustness study—five independent initial condition sets all produce S-boxes satisfying: 0 fixed points, 0 two-cycles, NL ≥ 100, DU ≤ 12, confirming the robustness of the proposed construction across different chaotic initial conditions.

**Table 7:** Comparative table for cryptographic metrics vs. state of the art.

Method	NL	DU	Av%	Deg	FP	KeyDep
Ideal	112	4	50/0	7	0	-
AES S-box [23]	112	4	50.0	7	0	No
Logistic Map [49]	84	16	49.5	6	2	Yes
Lorenz-based [50]	96	10	49.8	7	1	Yes
COBLAH [13]	108	8	50.1	7	0	Yes
Hyperchaotic [19]	98	12	49.7	7	0	Yes
Elliptic Curve [14]	104	8	50.2	7	0	No
Proposed	104	8	50.66	7	0	Yes

NL = nonlinearity; DU = differential uniformity; Av% = average avalanche effect; Deg = algebraic degree; FP = number of fixed points; KeyDep = key-dependent S-box (dynamic). All metrics are computed for 8-bit → 8-bit bijective S-boxes. Av% ideal value = 50.00%.

**Table 8:** Summary of generated S-box.

Metric	Mean	Std	Min	Max
Nonlinearity (NL)	104.0	0.0	104	104
Differential uniformity (DU)	6.93	1.06	6	10

**Table 9:** Comparative values for BCT and AI analysis.

Metric	Proposed	AES	Interpretation
BCT maximum (boomerang uniformity)	16	6	Within the chaos-based range; no short-cycle structure for a boomerang distinguisher
Algebraic immunity (AI)	2	2	Equal to AES; lowest relation degree is 2

**Table 10:** Statistical results for the proposed algorithm for the grayscale image [256×256] in comparison to others.

Analysis	Images	Correlation	Entropy	Homogeneity	Contrast	Energy
[55]	Baboon	0.0112	7.9973	0.2315	7.8651	0.0101
[56]	Baboon	0.0336	7.8815	0.3452	6.8815	0.0128
[57]	Baboon	−0.0300	7.997	-	-	-
[31]	Lena	-	7.9891	-	-	-
Proposed	Baboon	0.0018	7.9992	0.1986	8.4217	0.0089
Proposed	Lena	0.0025	7.9987	0.2054	8.1123	0.0094

**Table 11:** Statistical results for the proposed algorithm for synthetic medical images.

Image	Entropy (Orig -> Enc)	NPCR	UACI	Corr (Orig -> Enc)
Synthetic CT	5.172 -> 7.997	100%	33.5%	0.960 -> −0.010
Synthetic X-ray	5.843 -> 7.997	100%	33.7%	0.979 -> 0.015
Ideal	8.000	≥99.60	≈33.46	0

**Table 12:** Serial monitor output.

(1) 23.3C 60.7% PT:17 03 3C 07 00 01 00 00 CT:81 31 65 61 4E 4E 9B 1D 128us PASS (2) 23.6C 61.4% PT:17 06 3D 04 00 02 00 00 CT:81 D4 F6 A0 4E 4F 9B 1D 128us PASS (3) 23.9C 62.1% PT:17 09 3E 01 00 03 00 00 CT:81 48 69 71 4E 0F 9B 1D 124us PASS (4) 24.2C 62.8% PT:18 02 3E 08 00 04 00 00 CT:2B 6D 69 FA 4E 75 9B 1D 128us PASS (5) 24.5C 63.5% PT:18 05 3F 05 00 05 00 00 CT:2B 7F 3C D3 4E 31 9B 1D 124us PASS (6) 24.8C 64.2% PT:18 08 40 02 00 06 00 00 CT:2B B6 C9 FD 4E 54 9B 1D 128us PASS (7) 25.1C 64.9% PT:19 01 40 09 00 07 00 00 CT:4B E2 C9 32 4E 22 9B 1D 124us PASS (8) 25.4C 65.6% PT:19 04 41 06 00 08 00 00 CT:4B F9 3F 94 4E 64 9B 1D 124us PASS (9) 23.2C 66.3% PT:17 02 42 03 00 09 00 00 CT:81 6D 07 62 4E 83 9B 1D 128us PASS (10) 23.5C 67.0% PT:17 05 43 00 00 0A 00 00 CT:81 7F 56 B2 4E 9A 9B 1D 128us PASS
---- Periodic Summary ----
Readings encrypted: 10 | Total data: 160 bytes | S-box cipher throughput: 126.58 KB/s | System throughput: 5 B/s (DHT22 sensor-rate limited not representative of cipher speed) | Free SRAM: 1434 bytes.

**Table 13:** Proposed S-box hardware performance on Arduino Uno (ATmega328P, 16 MHz), with AES-128 and PRESENT-80 as implementation-level baselines.

Metric	Proposed S-Box	AES-128 [60]	PRESENT-80 [59]
Block size (bytes)	16	16	8
Per-block latency (μs)	126.4	~2300	~232
Per-byte latency (μs)	7.90	~143.8	~29.0
Throughput (KB/s)	126.58	~6.96	~34.48
S-box Flash usage (bytes)	256	256	160
SRAM consumption (bytes)	614	~700	~200
SRAM headroom	70.0%	~65.8%	~90.2%
Correctness (10 readings)	10/10 PASS	N/A	N/A
Compressed half-table (bytes)	128	N/A	N/A
Compressed latency (μs)	142.0 (+12.3%)	N/A	N/A
**Resource**	**Usage**	**Share of ATmega328P budget**	
Forward S-box (Flash/PROGMEM)	256 B	0.8% of 32 KB	
Inverse S-box (SRAM, built once at startup)	256 B	12.5% of 2 KB	
Total SRAM (with buffers)	~614 B	30% of 2 KB (1434 B free)	
AES-128: Arduino C-port implementation PRESENT: Uses 8-byte blocks, throughput computed per respective block size			

**Table 14:** Summary of power consumption.

Cipher	Latency/16 B	Energy/Block	Avg. Encryption Power
Proposed (LUT)	126.4 μs	7.9 nJ	~3.9 μW
AES-128	~2300 μs	143.75 nJ	~72 μW
PRESENT-80	~230 μs	~14.4 nJ	~7.2 μW

## Data Availability

The data presented in this study are available within the article. Additional data supporting the reported results, including the generated S-box values, cryptographic evaluation outputs, image-encryption test results, and IoT hardware performance records, are available from the corresponding author upon reasonable request.

## Abbreviations

The following abbreviations are used in this manuscript:

S-box	Substitution Box
GF (2)	Galois Field of Order 2
SHA-256	Secure Hash Algorithm 256-bit
KDF	Key Derivation Function
NIST	National Institute of Standards and Technology
SP 800-22	Special Publication 800-22
LE	Lyapunov Exponent
DU	Differential Uniformity
SAC	Strict Avalanche Criterion
LAT	Linear Approximation Table
DDT	Difference Distribution Table
AES	Advanced Encryption Standard
ABC	Artificial Bee Colony
BFO	Bacterial Foraging Optimization
AJO	Artificial Jellyfish Optimization
GWO	Grey Wolf Optimizer
COBLAH	Chaotic Opposition-Based Learning Initialized Hybrid Algebraic–Heuristic
